# Roles of glucose‐dependent insulinotropic polypeptide in diet‐induced obesity

**DOI:** 10.1111/jdi.13816

**Published:** 2022-05-11

**Authors:** Yusuke Seino, Yuji Yamazaki

**Affiliations:** ^1^ Department of Endocrinology, Diabetes and Metabolism Fujita Health University Toyoake Japan; ^2^ Yutaka Seino Distinguished Center for Diabetes Research Kansai Electric Power Medical Research Institute Kobe Japan; ^3^ Center for Diabetes, Endocrinology and Metabolism Kansai Electric Power Hospital Osaka Japan

**Keywords:** Glucagon‐like peptide‐1, Glucose‐dependent insulinotropic polypeptide, Nutrients

## Abstract

Glucose‐dependent insulinotropic polypeptide (GIP) and glucagon‐like peptide‐1 (GLP‐1) are incretins that play an important role in glucose metabolism, by increasing glucose‐induced insulin secretion from pancreatic β‐cells and help regulate bodyweight. Although they show a similar action on glucose‐induced insulin secretion, two incretins are distinct in various aspects. GIP is secreted from enteroendocrine K cell mainly expressed in the upper small intestine, and GLP‐1 is secreted from enteroendocrine L cells mainly expressed in the lower small intestine and colon by the stimulation of various nutrients. The mechanism of GIP secretion induced by nutrients, especially carbohydrates, is different from that of GLP‐1 secretion. GIP promotes fat deposition in adipose tissue, and contributes to fat‐induced obesity. In contrast, GLP‐1 participates in reducing bodyweight by suppressing food consumption and/or slowing gastric emptying. There is substantial evidence that GIP and GLP‐1 might differently contribute to bodyweight control. Although meal contents influence both glycemic and weight control, we do not fully understand whether incretin actions differ depending on the contents of the meal and what kind of signaling is involved in its context. We focus on the molecular mechanism of GIP secretion induced by nutrients, as well as the roles of GIP in weight changes caused by various diets.

## Graphical abstract

The effects on pancreatic and gastrointestinal hormone secretion differ by the type of the nutrients ingested.

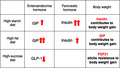

## Introduction

Incretins are released from enteroendocrine cells (EECs) by sensing orally ingested nutrients, and play pivotal roles in glucose and lipid metabolisms. GIP was the first identified incretin hormone, and then GLP‐1 was characterized as another incretin hormone^1^. Although these two incretin hormones show an insulinotropic action in a glucose‐dependent manner, GLP‐1 suppresses glucagon secretion, but GIP stimulates glucagon secretion as an intrapancreatic effect. GIP and GLP‐1 also exert various extrapancreatic actions in the adipose tissues, brain, bone, heart and so on^2^. These hormones also play an important role in regulating bodyweight. GLP‐1 reduces bodyweight by slowing gastric emptying and suppressing food intake by activating the hypothalamus and hindbrain GLP‐1 receptors, whereas GIP promotes nutrient uptake into adipose tissue. Unlike GLP‐1, plasma GIP levels rise immediately after oral carbohydrate and fat consumption, causing obesity in the event of an overdose. As a result, understanding the mechanism of GIP secretion by nutrients will pave the way for a treatment strategy for obesity and diabetes. Dietary differences are associated with differences in GIP secretion, as well as glycemic and weight control. The molecular mechanisms of GIP secretion induced by various diets are discussed here, as well as the physiological roles of GIP in our bodies.

## The Distinct Distribution of EECS Producing GIP and GLP‐1, Respectively

GIP and GLP‐1 are secreted by different subtypes of EECs, implying that the timing of their secretion and nutrient sensing differ^3^. Each subtype of EECs is distributed with a distinct gradient from the duodenum to the ileum in the gastrointestinal tract, implying a distinct nutrient sensing and role^4^. Traditionally, EECs were thought to secrete each specific gut hormone. As for incretins, K and L cells secrete GIP and GLP‐1, respectively. Later, some portions of EECs were found to secrete both GIP and GLP‐1, the so‐called K/L cells, which is consistent with the large heterogeneity of EECs described by recent single‐cell ribonucleic acid sequencing analyses^5^. Rather than the distribution of K and L cells, it is relevant that the number of GIP‐producing cells is the highest in the duodenum, and gradually decreases in the lower small intestine, whereas the number of GLP‐1‐producing cells is relatively lower in the upper intestinal tract and highest at the ileum terminal. Consistent with their distribution, GIP signaling accounts for two‐thirds of incretin effects in healthy organisms, and then, the rest of the incretin effects are derived by GLP‐1 signaling^6^. GIP signaling seems to be equipped for the early response to food intake.

## The Molecular Mechanisms of GIP Secretion are Regulated by Sugars in the Intestinal K Cells

GIP secretion is directly induced by carbohydrates and lipids in intestinal K cells, which is strongly linked to nutrient sensing at the apical side of EECs. Among carbohydrates, glucose has a strong stimulatory effect on both GIP and GLP‐1 secretion. Sodium–glucose cotransporter 1 (SGLT1) transports glucose from the intestinal lumen into the intestinal cytosol in intestinal absorptive cells. SGLT1 is also located in luminal membranes of both K and L cells^7^. Glucose‐induced GIP secretion is completely blocked in SGLT1‐deficient mice and in mice treated with the SGLT1 inhibitor, phlorizin^7^, ^8^, indicating that glucose‐induced GIP secretion is SGLT1‐dependent. On the contrary, in SGLT1‐deficient mice and mice treated with a dual SGLT1 and SGLT2 inhibitor, plasma GLP‐1 levels during the oral glucose tolerance test are completely blocked at 5 min after glucose loading, but remain high thereafter^7^, ^9^, suggesting that there is an SGLT1‐independent pathway for GLP‐1 secretion by glucose.

The sweet substance binds to the sweet receptor (T1R2/T1R3) and activates signal transduction, including α‐gustducin. Glucose‐induced GLP‐1 secretion, but not GIP secretion, is impaired in α‐gustducin‐deficient mice^10^. In addition, another monosaccharide, fructose, stimulates GLP‐1 secretion, but not GIP secretion in mice, rats and humans^11^. These facts might suggest that GLP‐1 secretion, but not GIP secretion, by glucose and fructose is induced through the sweet taste signal.

**Figure 1:**
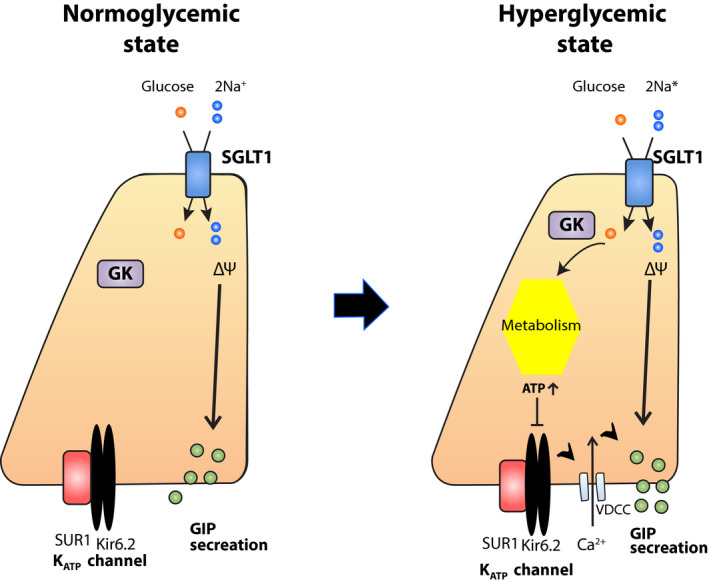
Mechanism of glucose‐dependent insulinotropic polypeptide (GIP) secretion induced by glucose in K cells. Under the normoglycemic state, glucose loaded in the intestinal lumen is transported through sodium–dependent glucose cotransporter 1 in the apical side of K cells, which induces membrane depolarization and GIP secretion. Adenosine triphosphate‐sensitive potassium (K_ATP_) channels are expressed in K cells. However, they remain closed in the basal condition. Under the high glycemic state, K_ATP_ channels maintain open. When glucose is transported, the elevation of cellular ATP levels caused by cellular metabolism leads to close K_ATP_ channels, which also triggers membrane depolarization, and involves in GIP secretion in addition to the sodium–dependent glucose cotransporter 1 (SGLT1)‐dependent manner. FGF21, fibroblast growth factor 21; GK, glucokinase; SUR1, sulfonylurea receptor 1; VDCC, voltage‐dependent Ca channel. [Colour figure can be viewed at wileyonlinelibrary.com]

Interestingly, glucose‐induced GIP secretion is enhanced, and fructose significantly stimulates GIP secretion in streptozotocin‐induced diabetic rodents^8^, ^12^, ^13^. Adenosine triphosphate‐sensitive potassium (K_ATP_) channels, which consist of Kir6.2 and sulfonylurea receptor 1 (SUR1) subunits, play an essential role in glucose‐induced insulin secretion (GIIS) in pancreatic beta cells. The K_ATP_ channels are expressed in purified K cells^14^. We propose the model shown in Figure 1 ^15^. In a normoglycemic state, K_ATP_ channels on the lateral side of K cells are closed, and glucose transported through SGLT1 causes membrane depolarization, which drives GIP secretion. Under a hyperglycemic state, the K_ATP_ channels open in basal conditions. When glucose is loaded, an increase in adenosine triphosphate concentration closes the K_ATP_ channels, depolarizing the membrane and augmenting SGLT1‐dependent GIP secretion. On the contrary, K_ATP_ channels do not contribute to fructose‐induced GIP secretion under hyperglycemic conditions^13^. The molecular mechanism of fructose‐induced GIP secretion remains unclear. Additionally, it would be interesting to investigate whether fructose metabolism in a hyperglycemic state might enhance the secretion of either gut‐derived GIP or islet‐derived GIP based on the expression of glucose transporter 5 for fructose transportation in both the intestinal K cells and pancreatic alpha cells.

## Energy Balance is Differently Affected by the Intake of Various Carbohydrates

Total daily caloric intake is generally recommended for bodyweight management. In Japan, a protein, fat and carbohydrates balanced diet consists of 40–60% carbohydrates, approximately 20% proteins and 20–30% fat. Many clinical studies on the relationship of carbohydrate intake to obesity fail to mention the wide range of carbohydrate types, although different carbohydrate types might be associated with different phenotypes of weight gain and glucose metabolism.

We investigated the effect of a high‐starch diet, which is metabolized into glucose, and a high‐sucrose diet, which is metabolized into glucose and fructose on change in bodyweight and glucose metabolism using mice^16^. Despite the same high‐carbohydrate and calorie content, a high‐starch diet results in greater weight gain; in contrast, a high‐sucrose diet containing 38.5% solid sucrose provides resistance against obesity. Fibroblast growth factor 21 (FGF21) is mainly produced from the liver, and increases energy expenditure in white and brown adipose tissues. We clarified that energy expenditure in mice fed a high‐sucrose diet significantly increased compared with mice fed a standard or high‐starch diet, due to increased FGF21 production both in the liver and adipose tissue, which contributes to the resistance to weight gain in mice fed a high‐sucrose diet^16^.

**Figure 2:**
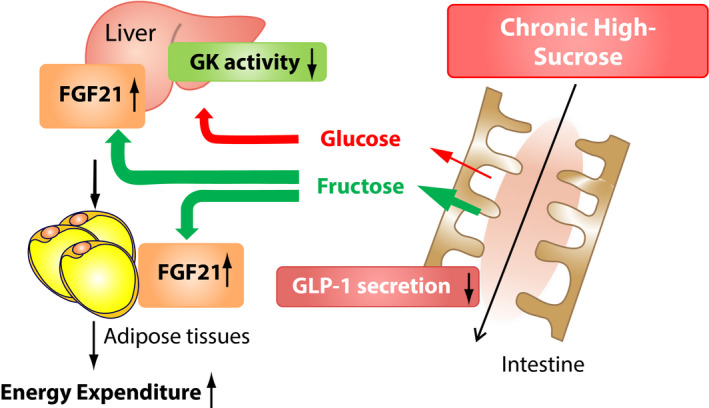
Effect of chronic high‐sucrose diet on bodyweight glucose metabolism. Chronic ingestion of a high‐sucrose diet would induce an increased amount of sucrose reaching the liver through the portal vein, which leads to increased fibroblast growth factor 21 (FGF21) in the liver and adipose tissues. FGF21 enhances the energy expenditure in adipose tissue. Although a chronic high‐sucrose diet might contribute to less bodyweight gain, it induces the decrease of glucokinase (GK) activity, and glucagon‐like peptide‐1 (GLP‐1) secretion, which might weaken glucose tolerance. [Colour figure can be viewed at wileyonlinelibrary.com]

Although the virtue of a high‐sucrose diet is resistance to bodyweight gain, mice fed a high‐sucrose diet show glucose intolerance due to the decrease of glucokinase activity in the liver and GLP‐1 secretion involving downregulation of sweet taste receptors^17^ (Figure 2). In addition, it has been reported that mice drinking liquid sucrose show bodyweight gain, but not mice fed the same amount of solid sucrose as a result of greater energy intake probably due to low satiety of liquid sucrose^18^. Therefore, a high‐sucrose diet, let alone liquid sucrose, seems not to be recommended for bodyweight management.

What is an appropriate amount of sucrose/fructose consumption recommended for bodyweight management? According to the Australian Diabetes, Obesity and Lifestyle Study, increased fruit consumption reduced the risk of developing type 2 diabetes over a 5‐year follow‐up period due to an increase in insulin sensitivity in healthy individuals^19^. Several potential mechanisms are considered to reduce the risk of type 2 diabetes through fruit consumption^20^. Fruits are generally high in fructose, fiber and phytochemicals (flavonoids). Fiber makes glycemic load slow down and maintains preferable intestinal flora. Flavonoids might have good effects on insulin sensitivity. Fructose stimulates FGF21 production in the liver and GLP‐1 secretion in the gut. As indicated before, FGF21 increases energy expenditure. In addition, FGF21 and GLP‐1 play a significant role in satiety in the brain. As a result, fructose might be an important nutrient in bodyweight control, leading to an increase in insulin sensitivity. In contrast, epidemiological studies show a strong link between high fructose intake and obesity and non‐alcoholic fatty liver disease, which is consistent with the fact that fructose consumption has skyrocketed over the past two centuries^21^, ^22^. Excess fructose intake overwhelms intestinal fructose clearance, resulting in toxic fructose exposure on the liver, which causes fatty liver and obesity^23^. As the fructose metabolism has recently been unveiled, further investigation is required for understanding how much fructose intake would be permitted for our health.

## GIP is Associated with Fat‐induced Obesity (Not in Starch‐induced Obesity)

It is well characterized that GIP directly and in collaboration with insulin promotes fat accumulation. Excess fat and carbohydrate intake is also well known to cause obesity concomitant with hyperinsulinemia and hyper‐GIP secretion in mice and humans. Among patients with type 2 diabetes treated with dipeptidyl peptidase‐4 (DPP‐4) inhibitors, which are supposed to enhance GIP action, patients who gained bodyweight and worsened glycemic control showed excessive intake of fat, but not carbohydrate, compared with those who maintained bodyweight with good glycemic control^24^. In contrast, GIP receptor‐deficient mice fed a high‐fat diet did not show obesity, but those fed a high‐starch diet showed bodyweight gain, the ratio of which is almost the same as that in wild‐type mice, although both types of diet‐induced bodyweight gain in wild‐type mice^25^. These results suggest that obesity induced by excess fat intake appears to substantially rely on hyper‐GIP secretion, which is different from high‐starch‐induced obesity (Table 1).

**Table 1:** Effects on pancreatic and gastrointestinal hormone secretion differ by the type of the nutrients ingested

	Enteroendocrine hormone	Pancreatic hormone	Bodyweight
High‐starch diet	GIP 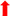	Insulin 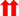	Insulin contributes to bodyweight gain
High‐fat diet	GIP 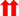	Insulin 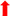	GIP contributes to bodyweight gain
High‐sucrose diet	GLP‐1 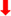		FGF21 elicits resistance to bodyweight gain

FGF21, fibroblast growth factor 21; GIP, glucose‐dependent insulinotropic polypeptide; GLP‐1, glucagon‐like peptide‐1.

The fact that GIP participates in fat accumulation in adipose tissue implied that bodyweight gain due to excess fat intake would be caused by enhanced fat storage in adipose tissue by GIP signaling. However, adipose tissue‐specific GIP receptor‐deficient mice do not mitigate the gain of bodyweight or fat mass caused by a high‐fat diet^26^, ^27^. In contrast, GIP receptors are also expressed in the brain, and the intracerebroventricular administration of GIP receptor‐antagonizing antibodies suppressed the weight gain of mice fed with a high‐fat diet due to a decreased food intake induced by enhanced leptin sensitivity^28^. Indeed, when fed with a high‐fat diet, these mice treated with the antagonizing antibody of GIP receptor or GIP receptor‐deficient mice showed reductions in food intake and adipose tissue weight^25^, ^29^. Thus, GIP signaling in the brain might be a key to understand how hyper‐GIP secretion (derived from an endogenous hyper‐GIP secretion) induces bodyweight gain.

It is generally recognized that insulin is involved in bodyweight gain through hyperinsulinemia or insulin resistance. When insulin sensitivity decreases, GIP plays an essential role in regulating bodyweight^30^. In mice fed with a high‐fat diet, insulin sensitivity was significantly decreased based on the insulin tolerance test, and the insulin secretory response to GIP was enhanced with the increased messenger ribonucleic acid expression levels of total GIP receptors in isolated islets compared with that in mice fed with a high‐starch diet^25^. This suggests a clear difference in the involvement of GIP signaling between a high‐fat diet and a high‐starch diet.

Obesity caused by a high‐carbohydrate diet is primarily dependent on hyperinsulinemia. Carbohydrate consumption significantly increases insulin secretion, including cephalic‐phase insulin secretion mediated by parasympathetic nerves. Furthermore, due to increased glucose supplementation, chronic consumption of a high‐starch diet causes β‐cell mass expansion^31^. These results show that the action of insulin is sufficient to control bodyweight of mice fed with a high‐starch diet, but not that of mice fed with a high‐fat diet.

Accumulated evidence suggests that pancreatic and enteroendocrine hormone secretions and the impact on bodyweight management differ by the content of ingested carbohydrates and fat. Further studies are required in healthy individuals and patients with diabetes.

## The Possible Role of Islet‐derived GIP and GLP‐1

Gut‐derived GIP and GLP‐1 are secreted from EECs in response to food ingestion, and potentiate GIIS as the classic incretin effect. Then, it is reported that GIP and GLP‐1 are found in pancreatic islets, which are called islet‐derived GIP and GLP‐1^32^, ^33^. The DPP‐4 inhibitor, anagliptin, improved glucose tolerance in normal glycemic wild‐type mice due to increased GIIS during the oral glucose tolerance test, which primarily stimulates gut‐derived GIP and GLP‐1, but not during the intraperitoneal glucose tolerance test, which primarily stimulates islet‐derived GIP and GLP‐1, showing that gut‐derived GIP and GLP‐1 are sufficient to maintain glucose metabolism under normal conditions^34^. Both glucagon and GLP‐1 are produced by the same precursor, proglucagon. In pancreatic α‐cells, glucagon is produced through cleavage of proglucagon by the enzyme prohormone convertase 2, and in intestinal L cells, GLP‐1 is produced through cleavage of proglucagon by prohormone convertase 1/3. Under the deficiency of glucagon action, especially in the liver and after vertical sleeve gastrectomy in rodents, islet‐derived GLP‐1 is produced due to the increase of prohormone convertase 1/3 expression levels in pancreatic α‐cells, leading to enhanced GIIS in a paracrine manner under normal conditions^35^, ^36^. In addition, it is reported that DPP‐4 is expressed in mouse and human islets, and that DPP‐4 inhibitors enhance GIIS from isolated islets of mice and humans by promoting the secretion of intact GLP‐1 from the islets^37^. Furthermore, islet‐derived GLP‐1 contributes to GIIS under metabolic stress, such as feeding a high‐fat diet and β‐cell damage treated with streptozotocin, which increased the demand of insulin secretion in mice^32^, ^38^. Thus, the effect of islet‐derived GLP‐1 on the enhancement of insulin secretion by glucose has been clarified in rodents.

Short‐form GIP (1–30) secreted from pancreatic α‐cells, in contrast, participates in insulin secretion by glucose in mice^33^. Mice deficient in proglucagon‐derived peptides, such as glucagon and GLP‐1, showed improved glucose tolerance due to increased GIIS by islet‐derived GIP concomitant with GIP expression in ectopic pancreatic β‐cells, an effect that is completely abolished in mice deficient in proglucagon‐derived peptides, such as glucagon and GLP‐1 and GIP receptor double deficient mice^39^. However, the physiological relevance of islet‐derived GIP under metabolic stress remains unclear, although GIP‐positive areas in pancreatic α‐cells and/or GIP contents in the pancreas are increased under the metabolic condition, such as β‐cell damage treated with low‐dose streptozotocin, insulin resistance and during pregnancy, and the DPP‐4 inhibitor, anagliptin, markedly improved glucose tolerance in mice deficient in proglucagon‐derived peptides, such as glucagon and GLP‐1, treated with streptozotocin during the intraperitoneal glucose tolerance test by the enhancement of GIIS without increasing β‐cell mass and pancreatic insulin contents^34^, ^40^, ^41^, ^42^.

Whether islet‐derived GIP and/or nutrient‐induced GLP‐1 contribute to regulating energy balance remains unknown.

## Future Direction

Excess carbohydrates or fat, as well as different types of carbohydrates, have been shown in mouse studies to contribute to obesity through distinct signaling networks. It is widely acknowledged that daily meal contents, as well as total calorie intake, might be important for bodyweight management. However, because daily meals greatly vary between countries and races, we do not have a simple answer for bodyweight management in humans. It would be beneficial to investigate the intervention with obesity‐related signaling pathways. Enhanced GIP signaling seems to be closely associated with fat‐induced obesity, independent of hyperinsulinemia. The Western pattern diet mostly contains red meat, high‐fat dairy products and high‐sugar foods, which would have significantly affected the obesity problem in Western countries. In contrast, Asian people consume more carbohydrates than Western people^43^, ^44^. As East Asian people are well known to show less insulin secretion than white people, hyperinsulinemia and GIP signaling would contribute to obesity differently between them. Therefore, it is important to accumulate evidence for the roles of GIP signaling in Asian people who are generally prone to have reduced β‐cell function rather than increased insulin resistance.

Furthermore, recent reports showed that GIP also directly regulates the feed center and induces satiety in mice^45^, ^46^. Exogenous GIP receptor agonists show reduced bodyweight due to decreased food consumption^46^, ^47^. Therefore, both the blockade of endogenous GIP signaling and exogenous enhanced GIP agonism reduced bodyweight by decreasing food intake, which is partly controversial in regard to the well‐characterized association of GIP with fat‐induced obesity. Further studies are warranted to understand GIP signaling in healthy and disease states.

In addition, the liver is an important organ for nutrient metabolism; glucagon and insulin regulate glucose, amino acid, and lipid metabolism in the liver. Therefore, it is important to elucidate the relationship between glucagon and insulin action and incretin secretion from the aspect of glucose, amino acid, lipid homeostasis and food intake in various pathological models.

## Disclosure

The authors declare no conflict of interest.
